# The Impact of *spgM*, *rpfF*, *rmlA* Gene Distribution on Biofilm Formation in *Stenotrophomonas maltophilia*

**DOI:** 10.1371/journal.pone.0108409

**Published:** 2014-10-06

**Authors:** Chao Zhuo, Qian-yu Zhao, Shu-nian Xiao

**Affiliations:** State Key Laboratory of Respiratory Diseases, the first affiliated hospital of Guangzhou Medical College, Guangzhou, China; Indian Institute of Science, India

## Abstract

**Background:**

*Stenotrophomonas maltophilia* is emerging as one of the most frequently found bacteria in chronic pulmonary infection. Biofilm is increasingly recognized as a contributing factor to disease pathogenesis. In the present study, a total of 37 isolates of *S. maltophilia* obtained from chronic pulmonary infection patients were evaluated to the relationship between biofilm production and the relative genes expression.

**Methods:**

The clonal relatedness of isolates was determined by pulse-field gel electrophoresis. Biofilm formation assays were performed by crystal violet assay, and confirmed by Electron microscopy analysis and CLSM analysis. PCR was employed to learn gene distribution and expression.

**Results:**

Twenty-four pulsotypes were designated for 37 *S. maltophilia* isolates, and these 24 pulsotypes exhibited various levels of biofilm production, 8 strong biofilm-producing *S. maltophilia* strains with OD492 value above 0.6, 14 middle biofilm-producing strains with OD492 average value of 0.4 and 2 weak biofilm-producing strains with OD492 average value of 0.19. CLSM analysis showed that the isolates from the early stage of chronic infection enable to form more highly structured and multilayered biofim than those in the late stage. The prevalence of *spgM*, *rmlA*, and *rpfF* genes was 83.3%, 87.5%, and 50.0% in 24 *S. maltophilia* strains, respectively, and the presence of *rmlA*, *spgM* or *rpfF* had a close relationship with biofilm formation but did not significantly affect the mean amount of biofilm. Significant mutations of *spgM* and *rmlA* were found in both strong and weak biofilm-producing strains.

**Conclusion:**

Mutations in *spgM* and *rmlA* may be relevant to biofilm formation in the clinical isolates of *S. maltophilia*.

## Introduction

*Stenotrophomonas maltophilia* is a Gram-negative opportunistic pathogen in hospitalized or compromised patients [1], [2]. However, the role of this opportunistic pathogen as an innocent bystander or causative agent often remains unclear [3], [4] and little is known about its virulence factors [5], [6].

Biofilms, sessile structured bacterial communities exhibiting recalcitrance to antimicrobial compounds and persistence despite sustained host defenses, are increasingly recognized as a contributing factor to disease pathogenesis in the respiratory tract diseases associated with chronic bacterial infections [7]. *S. maltophilia* isolates are known to have the ability to form biofilms on both abiotic surfaces [8]–[10], and it is an intrinsic difference in biofilm formation among clinical isolates of *S. maltophilia*.

The molecular mechanisms underlying biofilm formation in *S. maltophilia* have not been extensively studied. Recently, mutants for *rmlA* gene and *rpfF* gene are reported to decrease biofilm formation [11], [12]. Further, the *spgM* gene, encoding a bifunctional enzyme with both phosphoglucomutase (PGM) and phosphomannomutase activities, could be involved in biofilm-forming ability because of the homology with the *algC* gene that is responsible for the production of a PGM associated with LPS and alginate biosynthesis in *P. aeruginosa* [13].

However, it is still unclear that which gene mutation results in the change of biofilm formation among the three genes, and which key amino acid mutation determines the function changes of the protein.

In this study, we examined a set of 24 clinical isolates of *S. maltophilia* for biofilm formation traits in order to find significant differences, and evaluated the relationship between biofilm production and the detection of *rmlA*, *spgM*, and *rpfF* genes.

## Materials and Methods

### Ethics Statement

This study protocol was approved by the Ethics Committee of The First Affiliated Hospital of Guangzhou Medical University. All subjects signed written informed consent prior to the study. Patient information was anonymized and de-identified prior to analysis.

### Bacterial isolates and growth conditions

Overall, 37 *S. maltophilia* isolates and *S. maltophilia* ATCC13637 were investigated. All the strains collected from the sputa of patients attending the Guangzhou institute of respiratory diseases from 2010 to 2011. Among the 37 isolates, 4 sequential strains isolated from the same patient over a period of 2 years and 3 sequential strains isolated from another patient over a period of 1 year were investigated. The isolates were identified as *S. maltophilia* by biochemical tests using manual (API 20-NE System; BioMérieux, Marcy-L'Etoile, France) or automated (Vitek; BioMérieux) systems, then stored at −80°C until use when they were grown at 37°C in Mueller-Hinton agar (MHA; Oxoid) plates.

### Genetic relatedness by PFGE and cluster analysis

Pulsed-field gel electrophoresis (PFGE) analysis of *Xba*I-digested genomic DNA was performed to determine the genetic relatedness of *S. maltophilia* isolates using a CHEF-Mapper XA System (Bio-Rad Laboratories, Hercules, CA, USA) as described by Seifert [14]. The interpreting criteria were described by Tenover [15] combining UPGMA (unweighted pair group method with hierarchic averages) method, Isolates were assigned the same pulsetype if the value of Dice coefficient of similarity was >80% [16].

### Biofilm formation assay

Overnight cultures in TSB were corrected with fresh TSB to an OD550 of 1.00 (corresponding to about 1×10^9^ CFU/ml). Two-hundred microliters of 1∶100 diluted inoculum were dispensed to each well of a sterile flatbottom polystyrene tissue culture 96-wells microtiter and incubated at 37°C for 24 h. Biofilm biomass was then measured by crystal violet assay. Briefly, biofilm samples were fixed for 1 h at 60°C, stained for 5 min at RT with 200 µl Hucker-modified crystal violet, then rinsed in standing water and allowed to dry. Biofilm samples were stained with 250 µl of 33% glacial acetic acid for 15 min, and the optical density at 492 nm (OD492) was read. Considering a low cut-off (ODc) represented by 3×SD above the mean OD of control wells, strains were classified into the following categories: no biofilm producer (OD≤ODc), weak biofilm producer (ODc<OD≤2×ODc), moderate biofilm producer (2×ODc<OD≤4×ODc), and strong biofilm producer (4×ODc<OD) [17].

The biofilm microstructure was detected by SEM and CLSM. SEM analyses were carried out with *S. maltophilia* strain S1219 and ATCC13637, selected because S1219 was the highest producer of slime among 24 strains considered. Biofilms were allowed to grow on cover slip, and fixed with 2.5% glutaraldehyde for at least 40 min. After being washed with PBS, the samples were then dehydrated in a series of aqueous ethanol solutions (50 to 100%). The specimens were mounted on aluminum stubs with conductive carbon cement, allowed to dry for 3 h, and then coated with 15-nm Au film with an agar automatic sputter coater. After processing, samples were observed with a QUANTA 200 scanning electron microscope in the high-vacuum mode at 15 kV.

CLSM was performed as pervious report [18]. Briefly, Biofilm samples were fixed in 2.5% glutaraldehyde, and stained with FITC (fluorescein isothiocyanate). CLSM analysis was performed with an LSM 510 META laser scanning microscope attached to an Axioplan II microscope. The excitation wavelengths were 458 [Argon laser], and 543 nm [He-Ne laser], and emission wavelengths were 488 nm for FITC, respectively. Depth measurements were taken at regular intervals across the width of the device. To determine the structure of the biofilms, a series of horizontal (x-y) optical sections were taken throughout the full length of the biofilm. Confocal images of green (FITC) fluorescence were conceived using a track mode.

### PCR-based genotyping for *rmlA*, *spgM*, and *rpfF*

Bacterial DNA was extracted and purified, then amplified and visualized on 2% agarose gel. PCR primers were respectively 5′ CGGAAAAGCAGAACATCG 3′ and 5′ GCAACTTGGTTTCAATCACTT 3′ (1222 bp) for *rmlA*, 5′ ATACCGGGGTGCGTTGAC 3′ and 5′ CATCTGCATGTGGATCTCGT 3′ (2750 bp) for *spgM* and, finally, 5′ CACGACAGTACAGGGGACC 3′ and 5′ GGCAGGAATGCGTTGG 3′ (1140 bp) for *rpfF*. All PCRs products were sequenced and BLAST in PUBMED. Furthermore, the three gene expression was performed with Reverse transcriptase PCR according to pervious report [18].

### Statistical analysis

All assays were carried out in triplicate and repeated twice, and the results are presented as means±SDs. The Pearson's correlation coefficient was calculated to determine the association between two variables. All P-values were based on two-tailed tests of significance, and a significance level of 0.05 or 0.01 was used. Statistical analysis of results was conducted with statistical software SPSS17.0.

## Results

### Clonal relatedness of isolates

**Figure 1:**
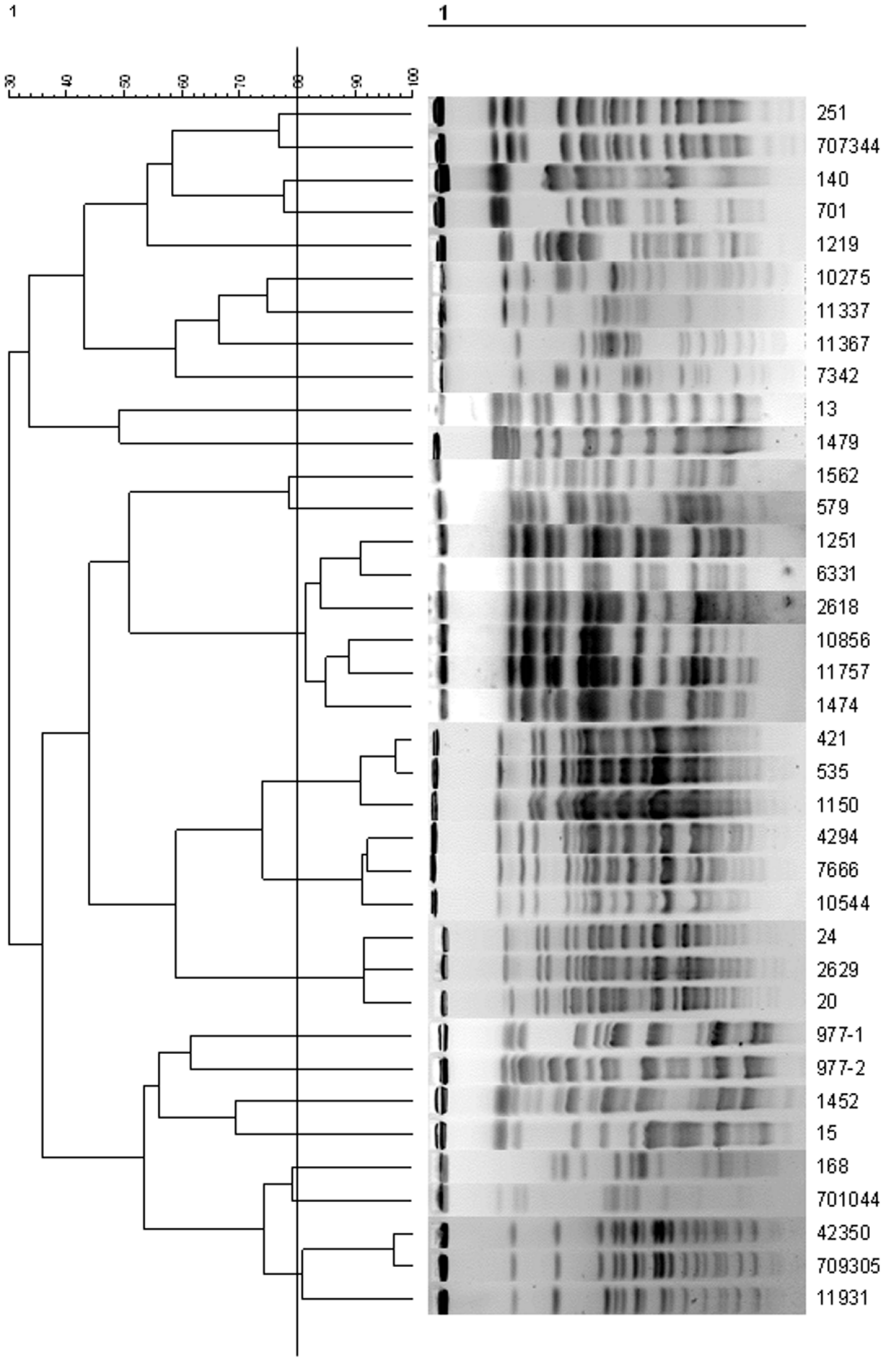
Dendrogram illustrating the percentage similarity of PFGE profiles in tested isolates of *S. maltophilia*.

According to the patterns of PFGE isolates, 24 pulsotypes were designated for 37 *S. maltophilia* isolates. Besides 19 individual pulsotypes, the remaining 18 isolates were classified into 5 pulsotypes, designated types A, B, C, D and E (Figure 1). Three out of 4 sequential strains isolated from one patient belong to type A, and the remaining 1 strain belong to one individual pulsotype (type T). Three sequential strains isolated from another patient belong to the same clone—type C. All the strains with individual pulsotypes and the 7 sequential strains were applied in further analysis.

### Ability of the isolates of *S. maltophilia* in forming biofilm

**Figure 2:**
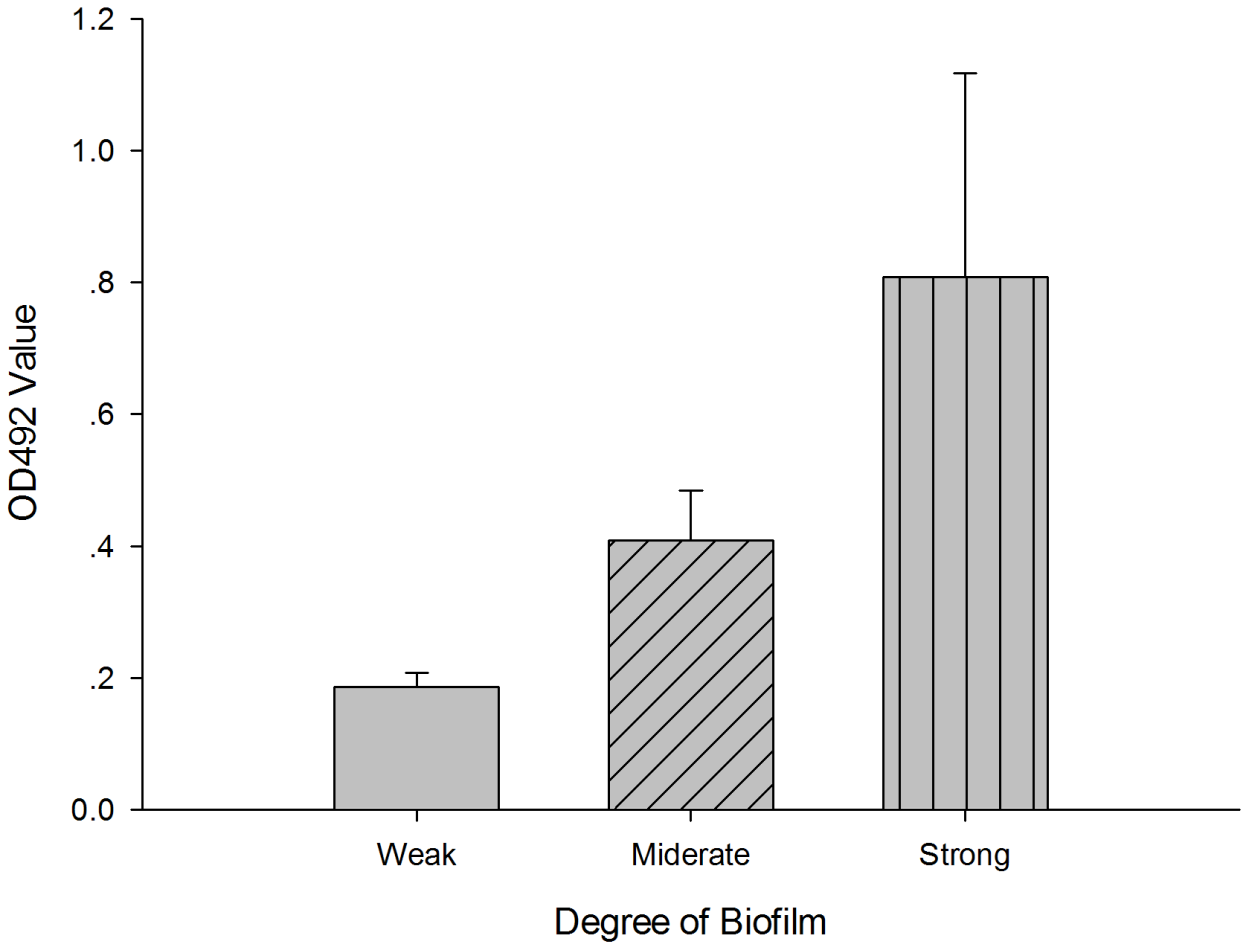
Histogram illustrating the ability of biofilm formation in tested isolates of *S. maltophilia*.

All the 24 isolates of *S. maltophilia* were able to form biofilm, and the biofilm forming ability varied greatly among strains tested (OD492 range: 0.18–1.4). Eight isolates had higher ability of biofilm-producing (OD492, mean ± SD: 0.808±0.310), 14 had middle (OD492, mean ± SD: 0.408±0.215) and 2 had lower (OD492, mean ± SD: 0.186±0.036) (Figure 2).

**Figure 3:**
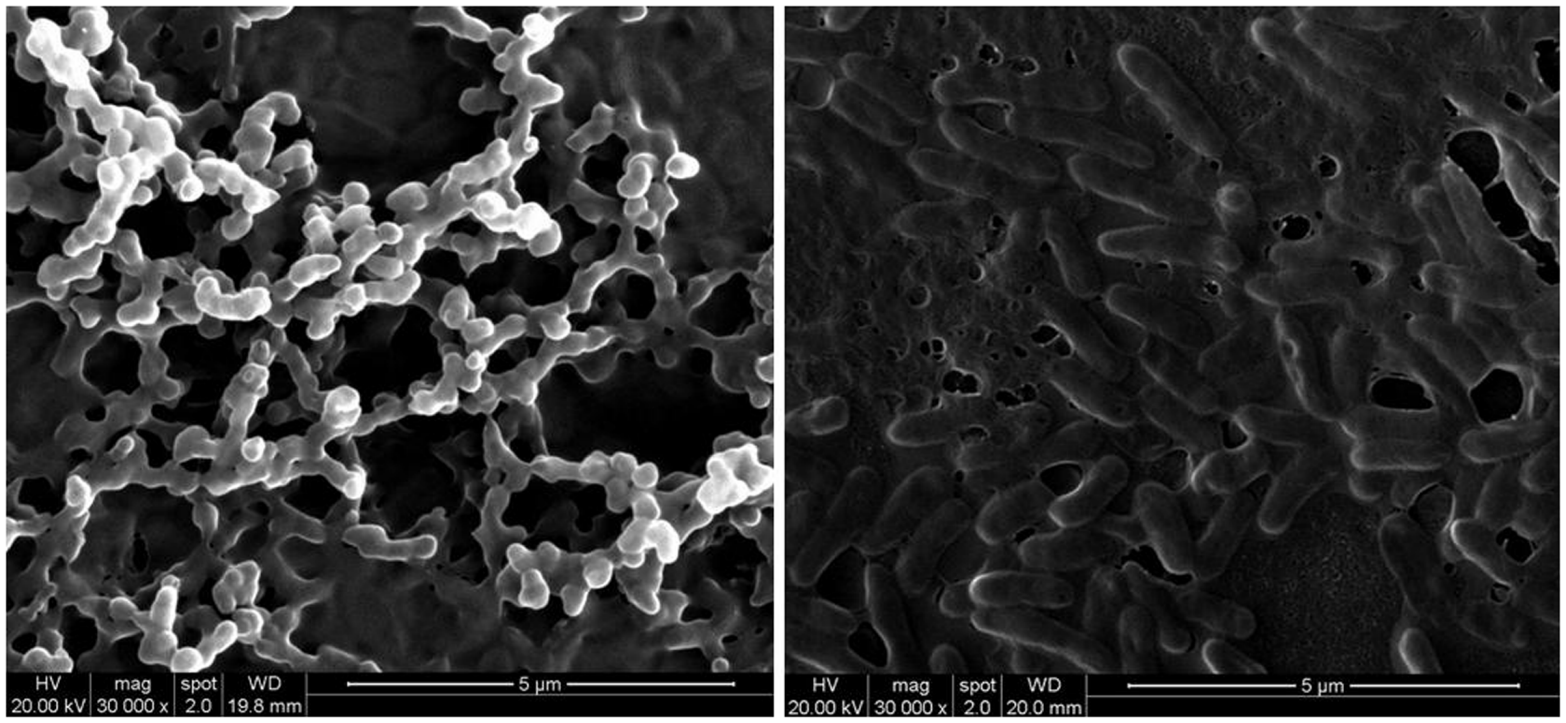
SEM of the strain Sm1219 of *S. maltophilia* biofilm mode in 8 h (left) and 24 h (right).

**Figure 4:**
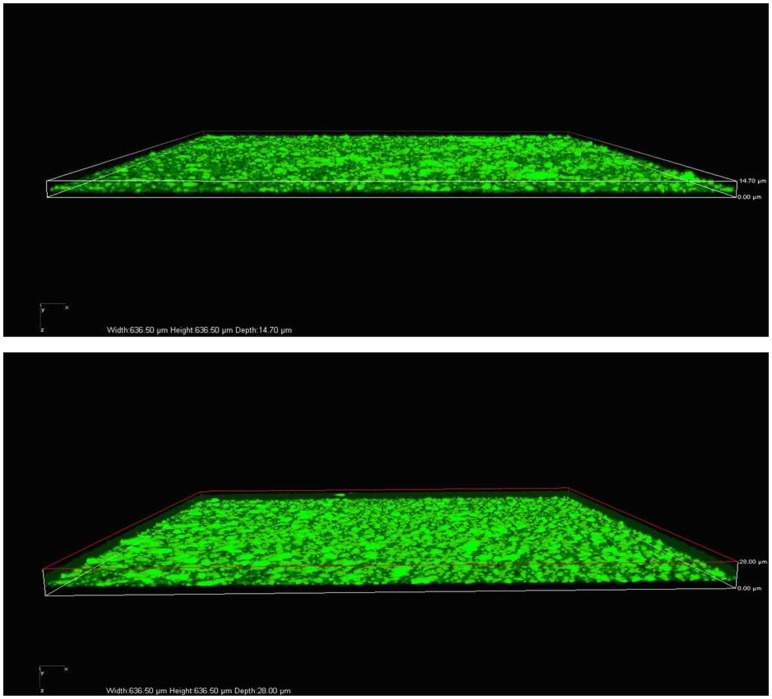
CLSM of the strain Sm1219 of *S. maltophilia* biofilm mode in 8 h (up) and 24 h (down).

Scanning electron micrographs (SEM) of the *S. maltophilia* biofilm mode of growth on polystyrene surface over 24 h of incubation was shown in figure 3. The biofilm covered just 70% of the surface, growing in specific clusters of cells (microcolonies), and a dense network of cells deeply embedded in an extracellular matrix. Spectrophotometric results were confirmed by Confocal Laser Scanning Microscopy (CLSM) analysis in figure 4. The biofilm formed by Sm1219 strain resulting to be the most complex, revealing a multilayered cell structure (58–63 µm, depth) embedded in an abundant extracellular polymeric substance (EPS) compared with *S. maltophilia* ATCC13637. Moreover, CLSM analysis revealed that the biofilm structure of the *S. maltophilia* isogenic sequential strains was significantly different in various isolated time. The 3 isogenic sequential strains belong to type C pulsotype displayed different ability in biofilm-formation were isolated from 11 months among his hospitalization. The first and second isolates revealed stronger ability in biofilm-formation with 0.56 and 0.60 of OD492 values, and the third isolate had weak ability in biofilm-formation with OD492 values of 0.19. Similarly, the 4 isolates of the other patient shown that the first and second isolates belong to type A pulsotype presented moderate ability in biofilm-formation with OD492 values of 0.40 to 0.42, while the fourth belong to type A presented weaker ability in biofilm-formation with OD492 values of 0.28. The third isolates (individual pulsotype) shown weak ability in biofilm-formation with D492 values of 0.20. All the two groups displayed that the strain isolated in early stage shown strong or middle biofilm-producing while the weak biofilm-producing in late stage. CLSM analysis also showed more highly structured, multilayered biofilm in early stage.

### Different genotypes and gene mutation are associated to ability of biofilm formation

**Figure 5:**
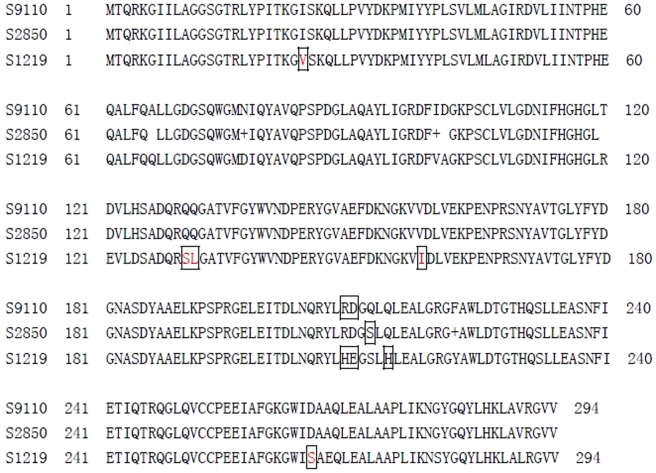
Analysis of rmlA amino acid mutation in isolates with vary ability of biofilm forming.

PCR-based typing of 24 *S. maltophilia* strains for *spgM*, *rmlA*, and *rpfF* genes showed an overall prevalence of 83.3%, 87.5%, and 50.0%, respectively. The presence of *rmlA*, *spgM* or *rpfF* had no close relationship with the ability of biofilm formation for each strain. However, considering the strain population as a whole, the presence of *spgM* and *rpfF* significantly improved biofilm formation. In particular, the strains with *spgM*+/*rpfF*+/*rmlA*+ genotype were easier to display strong or moderate biofilm-producer phenotype than the strains with *spgM*−/*rpfF−/rmlA+* genotype which was frequently detected in weak biofilm-producer phenotype (Table 1). Correlation analysis showed that the gene expression of *rmlA*, *spgM* and *rpfF* detected in the clinical strains were not associated to strong or moderate biofilm producers (For *rmlA*, Pearson r = 0.32, P>0.05; For *spgM*, Pearson r = 0.36, P>0.05; For *rpfF*, Pearson r = 0.23, P>0.05) (data not shown). In addition, significant amio acid mutation in *spgM* encoding gene and *rmlA* encoding gene were found between some strains of the strong and the weak biofilm-producer phenotype (Figure 5).

**Table 1:** The relation of different genotypes and the ability of biofilm formation in *Stenotrophomonas maltophilia* isolates with individual pulsotype.

NO. strains	Pulsotypes	OD492 values	Degree of BF	spgM	rpfF	rmlA
57-9110	G	0.17	weak	−	−	+
11931	E	0.20	weak	+	−	−
1150#	C	0.19	weak	−	−	+
1562*	T	0.20	weak	−	−	+
11757*	A	0.28	moderate	+	+	+
20	B	0.44	moderate	+	+	+
997	I	0.36	moderate	+	−	−
1251*	A	0.40	moderate	+	+	+
1474*	A	0.42	moderate	+	+	+
1452	M	0.38	moderate	−	−	+
15-2850	L	0.32	moderate	+	−	+
168	K	0.40	moderate	+	−	+
10544	D	0.50	moderate	+	+	+
701044	Q	0.40	moderate	+	−	+
251	S	0.31	moderate	+	+	−
707344	R	0.42	moderate	−	−	+
701	F	0.53	moderate	+	+	+
140	J	0.56	moderate	+	−	+
1479	O	0.37	moderate	+	+	+
421#	C	0.56	moderate	+	−	+
535#	C	0.60	strong	+	−	+
13	N	0.62	strong	+	+	+
997	V	0.63	strong	+	−	+
11337	W	0.65	strong	+	+	+
10275	H	0.68	strong	−	+	+
11367	U	0.68	strong	+	−	+
7342	H	1.20	strong	+	+	+
1219	P	1.40	strong	+	+	+

BF: Biofilm;

+: Gene expression in the *S. maltophilia* isolate;

−: No gene expression in *S. maltophilia*.

*: Four sequential strains isolated from one patient.

# : Three sequential strains isolated from another patient.

## Discussion

A significant feature of *S. maltophilia* is its ability to form biofilms on surfaces including Teflon, glass, and plastics and on host tissues and biofilms have been estimated to be associated with 65% of hospital-acquired infections [26]. However, it is not yet known whether there are any variations in biofilm formation among clonally diverse clinical isolates of *S. maltophilia*, and there are any relationships between biofilm forming ability and mutation or expression of biofilm-relative genes such as *spgM*, *rmlA*, and *rpfF* gene, although it was reported that the *spgM* mutant formed more biofilm than that formed by the parental strain on polystyrene microtiter wells and the *rmlA* and *rmlC* mutants produced significantly more biofilm on glass than that produced by the wild type [12], [25]. So, this study is designed to clarify these problems by analysis of morphology and genotype for biofilm-producing *S. maltophilia*.

Firstly, microtiter colorimetric assay for biofilm formation showed a wide range of biofilm formation ability, from biofilm-deficient phenotypes to those producing structurally complex biofilms. Morover, the images corresponded to three-dimensional reconstructions obtained from CLSM exhibited the native multicellular structures of biofilm in various forming ability, and CLSM analysis also showed that isolates from the early periods of chronic infection were able to form uniform flat biofilms or highly structured, multilayered and exopolysaccharide matrix-encased biofilms. On the contrary, isolates recovered from the late phase of chronic infection showed a significant reduction in adherence, lacking ability to form a mature biofilm. The variations in biofilm formation among the same or different patient may contribute to survival environment change of the bacteria. It was reported that he reduced efficiency in forming biofilm could be the consequences of *S. maltophilia* adaptation to a stressed environment such as CF lung [19]–[21], and oxidative stress and anaerobic conditions existed in COPD patient were also the risk factors to cause biofilm-producer reduced [22], and the similar support was found in *P.aeruginosa* [23].

In the present study we also focused our efforts on the relationship between biofilm formation and the presence of *rpfF*, *rmlA* and *spgM* genes. Overall, our results revealed the presence of *spgM* and *rpfF* significantly improved biofilm formation. The result was identified with Pompilio'report, which showed that *spgM* gene played a central role in biofilm formation of *S. maltophilia*, whose presence is significantly associated to a strong biofilm formation [9]. However, there were no regular mutation of *spgM* and *rmlA* gene between the strong and the weak biofilm formation in this study. Especially, 10 sites of amino acid mutation in *spgM* were found between the different phenotypes. *spgM* gene was shown to encode a bifunctional enzyme with both PGM and phosphomannomutase activities, an analysis of *S. maltophilia spgM* transposon insertion mutant strain JB12-23 showed that it formed more biofilm than that formed by its parental wild-type strain [13]. Unfortunately, it is uncertain that which site mutation in spgM might play a key role in the function change of SpgM by these reports. Similarly, the situation also appeared in *rmlA* gene. In this study, the *rmlA* genes shown more mutation sites than spgM gene among the clinical isolates with the three kinds of ability in biofilm formation, and only 15% amino acid sequences keep the homology between strong and weak ability groups. These results in this study may provide a clue for further study.

This study has several limitations. Firstly, it is difficult for us to observe dynamically the biofilm development of one strain such as adherence and twitching motility like previous report [23], [24], since the devices commonly used for the confocal study of biofilms, such as flow-cells, capillary tubes or glass coverslides, may be relatively expensive, which caused it difficult to observe dynamically the biofilm development of one strain such as adherence and twitching motility. Furthermore, we failed to analyze the relationship between ability of biofilm formation and key mutation of *rmlA* and *spgM* genes due to many variations in the amino acid sequence of the *rmlA* and *spgM* genes respectively. We try to find other better methods to understand the impact of these genes distribution on biofilm formation in *S. maltophilia* in further study.

In conclusion, the results showed that clinical strains of *S. maltophilia* significantly differ in some phenotype and genotype in biofilm formation, and also added new insight and expand previous knowledge concerning the relative genes.
